# Determinants of the Development of SARS-CoV-2 Anti-Spike Immune-Response after Vaccination among Healthcare Workers in Egypt

**DOI:** 10.3390/vaccines10020174

**Published:** 2022-01-22

**Authors:** Engy Mohamed El-Ghitany, Mona H. Hashish, Shehata Farag, Eman A. Omran, Azza Galal Farghaly, Nashwa Fawzy Abd El-Moez Azzam

**Affiliations:** 1 Department of Tropical Health, High Institute of Public Health, Alexandria University, Alexandria 21526, Egypt; azza.farghaly@alexu.edu.eg; 2 Department of Microbiology, High Institute of Public Health, Alexandria University, Alexandria 21526, Egypt; monash64@alexu.edu.eg (M.H.H.); Hiph.eomran@alexu.edu.eg (E.A.O.); HIPH.nazzam@alexu.edu.eg (N.F.A.E.-M.A.); 3 Department of Biostatistics, High Institute of Public Health, Alexandria University, Alexandria 21526, Egypt; babanada2010@alexu.edu.eg

**Keywords:** SARS-CoV-2 anti-spike antibody, healthcare workers, immune response, post-vaccination

## Abstract

Background: Understanding the factors affecting humoral immune response to COVID-19 vaccines among healthcare workers (HCWs) is essential to predict their level of protection. Vaccination elicits antibodies against SARS-CoV-2 spike protein (anti-S). Aim: To investigate the factors associated with the presence of SARS-CoV-2 anti-S antibodies among vaccinated HCWs. Methods: This cross-sectional study included 143 vaccinated HCWs, with or without a history of previous COVID-19 infection (clinically, radiologically, or by laboratory results) from different departments. Socio-demographic, clinical, as well as vaccine-related data, were recorded. Serum samples were collected and tested for SARS-CoV-2 spike antibodies. Results: Vaccination provoked an immunogenic response, where the overall anti-S positivity was 83.9% (95% CI: 77.8–90.0%). The response was not affected either by the age or gender of HCWs. Out of the 143 HCWs, 46 (32.1%; 95% CI: 24.4–39.9%) reported a previous history of COVID-19 infection, and seropositivity was significantly higher among them (*p* = 0.002), and it was associated with the frequency of infection (*p* = 0.044) and duration since diagnosis of COVID-19 infection (*p* = 0.065). They had higher median anti-S titers (111.8 RU/mL) than those without infection (39.8 RU/mL). Higher seropositivity was observed with Oxford/AstraZeneca vaccine (AZD1222) (88.9%; 95% CI: 83.1–95.0%) than Sinopharm (BBIBP-CorV) (67.7%; 95% CI: 50.3–85.2%), and with receiving two doses of vaccine (92.3%; 95% CI: 87.1–97.5%). Conclusions: Antibody positivity was significantly affected by the previous history of COVID-19 infection, type of vaccine, the number of doses received, and duration since vaccination.

## 1. Introduction

In the initial stage of coronavirus disease 2019 (COVID-19) pandemic, the World Health Organization (WHO) reported that 14% of COVID-19 cases were healthcare workers (HCWs) [1]. Globally, between 80,000 and 180,000 deaths occurred among HCWs between January 2020 and May 2021 [2]. Therefore, HCWs have had the priority for receipt of the COVID-19 vaccine [3].

Developing an effective immunity is the goal of all vaccines with different technologies. The humoral immune response is much easier to detect and far standardized than all other aspects of the immune response [4,5].

COVID-19 vaccines induce detectable humoral antibodies directed against different antigens of severe acute respiratory syndrome coronavirus 2 (SARS-CoV-2) [6]. One of the main immunogenic antigens in the post-vaccine immune response is the transmembrane spike (S) which is a receptor-binding domain (RBD) that protrudes from the viral surface and mediates viral entry into host cells [7]. However, the post-vaccination immune response and antibody titer remain markedly unpredictable, and many factors may affect it [8]. It had been reported to be age and gender-dependent, with higher titer in females and younger age groups [9]. Associated comorbidities that may weaken the immune response; such as cardiovascular disease, hypertension, diabetes, and obesity had been associated with lower levels of protective antibodies [10].

Additionally, vaccine type, number of doses and duration since the last vaccination markedly affect the immune response. Antibody level peaks two weeks after the second dose of vaccine (independently of age or gender) [11]. This study aimed to investigate the factors associated with the presence of SARS-CoV-2 anti-spike antibodies among vaccinated HCWs. In Egypt, vaccination started in March 2021. Initially, the Egyptian government made vaccines available to some HCWs, as well as vulnerable groups, including the elderly and people with chronic illnesses. Only two vaccines were available at that time; Sinopharm and AstraZeneca. The recommended dosages of AstraZeneca (ChAdOx1-S) were two doses given intramuscularly (0.5 mL each) with an interval of 8 to 12 weeks. Regarding Sinopharm (BBIBP-CorV) vaccine, they were 2 doses (0.5 mL) given intramuscularly at interval of 3–4 weeks. Currently, more vaccine types are available in Egypt, such as Pfizer, Moderna, Sinovac and Johnson & Johnson’s Janssen.

## 2. Method

This cross-sectional study was conducted throughout the period between January and June 2021. This period coincided with the second and third waves of the COVID-19 pandemic in Egypt. According to the Egyptian Ministry of Health and Population, the second pandemic wave of COVID-19 had stricken Egypt during the period from November 2020 till January 2021, and the third wave started from March 2021. During the second wave of infection, strains B.1.1.1. were sequenced [12].

### 2.1. Study Population

This study was a part of a project to study the seroprevalence of anti-S of SARS-CoV-2 among 2360 participants from the community and 559 HCWs from 39 hospitals from five governorates in Egypt. The study enrolled the vaccinated HCWs with or without a history of infection prior to vaccination (*n* = 143). Unvaccinated HCWs were excluded from the study. HCWs included physicians, nurses, technicians and pharmacists working at frontline departments (Intensive care units, emergency room, internal medicine wards, radiology, or laboratory) and low-risk departments (others). Criteria for diagnosis of previously infected cases (clinically, radiologically or by laboratory results) were according to the Egyptian national guidelines [13].

### 2.2. Data Collection

A structured interview questionnaire sheet was designed and filled in for each HCW, including all socio-demographic and clinical data. Height and weight of all HCWs were measured in order to calculate their Body Mass Index (BMI) according to the following formula; BMI = person’s weight in kilograms/ his height in meters squared (normal = 18.5–24.9, overweight = 25.0–29.9, obese = 30.0–39.9, and morbid obese >40). The smoking index was calculated for smokers (cigarette per day × years of tobacco use) and categorized into <400 and 400–800. Vaccine-related data as the type of vaccine, number of doses, and duration since the last dose were also recorded.

### 2.3. Laboratory Investigation

A three-ml venous blood sample was collected from each HCW and centrifuged at 3000 rpm. Serum samples were collected and stored at −20 °C until further processing.

The anti-SARS-CoV-2 Quantivac enzyme-linked immunosorbent assay(ELISA) (EuroImmun, Lübeck, Germany) was used for the quantitative detection of immunoglobulin class G (IgG) against the S1 domain of the viral spike protein. According to the manufacturer’s instructions, results should be interpreted according to their relative unit (RU) values as follows: <8 RU/mL were negative, while concentrations ≥8–<11 RU/mL were borderline and those ≥11 RU/mL were considered positive. Quantitative results were also expressed as quartiles since some samples had readings exceeding the highest calibrator in the kit (>120 RU/mL) and thus quartiles were used for correlations with quantitative variables. For comparing the results, the resulting concentrations in RU/mL were converted into BAU/mL by multiplying them by the factor of 3.2. The correlation analysis yielded a correlation coefficient of r = 0.99.

### 2.4. Ethical Considerations

The study was conducted in compliance with the Helsinki Declaration and was approved by the Institutional Review Board (IRB) Committee, Faculty of Medicine, Alexandria University; IRB number: 00012098- FWA number: 00018699, serial number:0305136. Administrative approval was taken from each healthcare setting prior to study onset. Anonymity and confidentiality were confirmed and written informed consent was obtained from each HCW.

### 2.5. Statistical Analysis

All statistical analysis was done using two-tailed tests. P-value less than 0.05 was statistically significant. Descriptive analysis based on frequency and percent distribution was done for all variables, including HCWs socio-demographic data and immunity status. Cross-tabulation was done to test some relations with serological findings among HCWs. Quantitative variables were expressed by the median and interquartile range (IQR), while categorical variables by absolute and relative frequency. The significance of relations was tested using the exact probability test for small frequency distribution. Initially, a statistical analysis plan that included all questionnaire data mentioned earlier was designed. However, because most of them did not significantly contribute to the risk of the primary outcome (detection of anti-S Ab), a multivariable model had been designed where variables were considered only if they were associated with the primary outcome in univariate analysis were selected in the final model.

## 3. Results

A total of 143 HCWs were vaccinated; the majority of them were women (55.24%), and ≥40 years (56.6%) (mean = 43.6, SD 11.1). Forty-six (32.17%) HCWs had a previous history of COVID-19 infection prior to vaccination.

Overall, anti- S seropositivity was detected among 120 (83.9%) out of 143 vaccinated HCWs (95% CI: 76.9–89.5%). The median anti-S titers among females was (68.4 RU/mL) and among males (53.5 RU/mL). No significant association was found between antibody response and age or gender of HCWs (*p*  = 0.172 and *p*  < 0.893, respectively). However, seropositivity was higher in both women and men <40 (88.7%) than those ≥40 (80.2%). Minor percentages of HCWs had comorbid conditions; 11.1% were diabetics, 22.3% were hypertensive, and 6.3% had other chronic conditions (asthma, allergy, cardiac, and renal diseases). None of these conditions was significantly associated with anti-S seropositivity. The majority of HCWs were overweight (41.9%), although not statistically significant, the highest anti-S seropositivity (89.5%) was observed among morbid obese HCWs, and the least was among those with normal BMI (76.9%). (Table 1).

**Figure 1:**
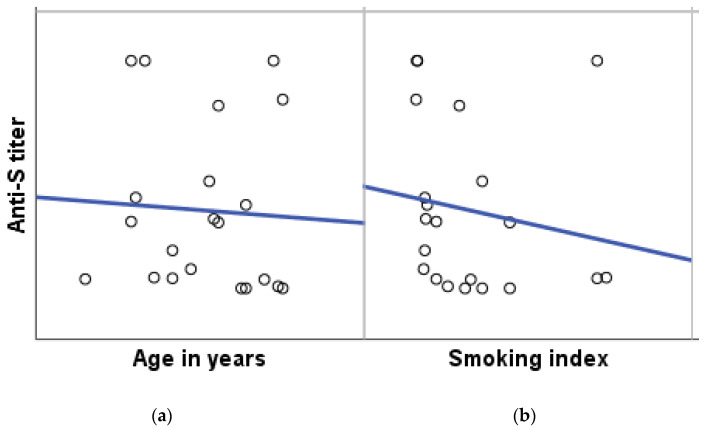
Correlation between anti-S titer and (**a**) the age in years and (**b**) the smoking index. Spearman’s correlation (rs) was used. rs with age= −0.16, *p*-value = 0.028 *; rs with smoking index (*n* = 21) = −0.431, *p*-value=0.026 *. * Correlation is significant at the 0.05 level (1-tailed).

Regarding the effect of smoking on post-vaccine antibody response, only 21 (14.7%) out of 143 HCWs were smokers. The post-vaccine antibody positivity was observed to be significantly lower in smokers (61.9%) than in non-smokers (87.7%) (*p* = 0.003). The correlation between anti-S titer and smoking index is illustrated in Figure 1.

Anti-S positivity was significantly higher in those who had COVID-19 infection before vaccination (97.8%) than those who had not (77.3%) (*p*  <  0.002), and it had been associated with the frequency of infection (*p* = 0.044) and duration since diagnosis (>3 months) (*p* = 0.065) (Table 2).

**Figure 2:**
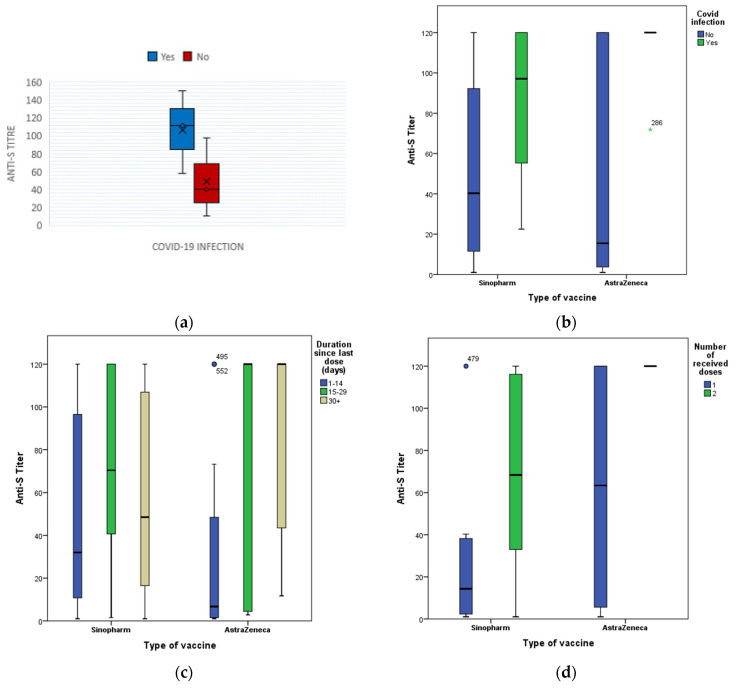
Box-Plot for anti-S titers (RU/mL) quartiles among vaccinated HCWs according to (**a**) the history of the previous infection, (**b**) the vaccine type in relation to the history of previous infection (**c**) the vaccine type in relation to the. duration since the last dose of vaccination and (**d**) the vaccine type in relation to the number of received doses. × indicates the mean value of the data being plotted. * indicates an outlier is present.

The difference in anti-S titers between samples from the previously infected participants (Median titer, 111.8; 95% CI, 75.7 to 150.0) and those from the previously uninfected (Median titer, 39.8; 95% CI, 24.0 to 69.2) (*p* < 0.001) is illustrated in Figure 2.

Out of 143 HCWs, 109 (76.2%) received Oxford/AstraZeneca vaccine (AZD1222), 31 (21.7%) received Sinopharm (BBIBP-CorV) vaccine and only 3 (2.1%) had other vaccines. HCWs who received Oxford/AstraZeneca vaccine (AZD1222) had a higher median anti-S titer (71.9 RU/mL) than those who received Sinopharm (58.0 RU/mL). Higher anti- S seropositivity (88.9%) was associated with Oxford/AstraZeneca vaccine (AZD1222) (*p* = 0.012), receiving two doses of vaccine (92.3%) (*p* = 0.001), and longer duration since last dose (≥14 days) (90%) (*p* = 0.43) (Table 3). Moreover, only 2 (6.5%) of the 31 HCWs during the study period had completed their two doses of Oxford/AstraZeneca vaccine (AZD1222), however, they showed a higher median anti-S titer after 14 days of vaccination than those receiving Sinopharm (BBIBP-CorV) vaccine, 99/109; 90.8% of them had completed their second dose (Figure 2).

**Figure 3:**
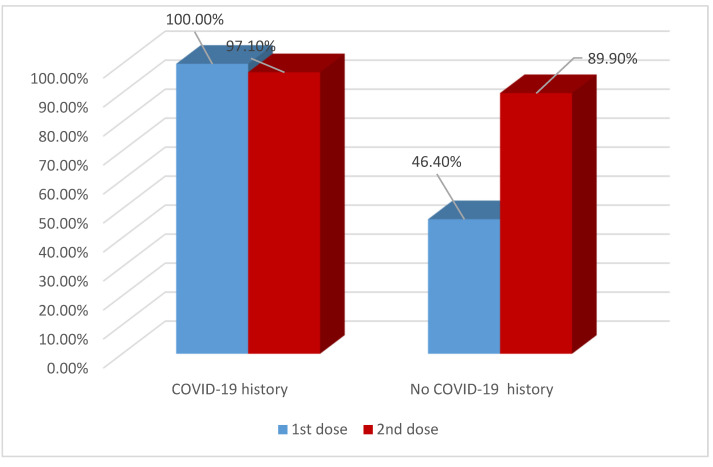
Anti-S seropositivity after each dose of vaccination according to COVID-19 history.

After the first dose of the vaccine, anti-S antibodies were detected in all 11 HCWs (100%) who had COVID-19 and in only 13 (46.4%) out of 28 HCWs who did not have COVID-19. (*p* = 0.002) (Figure 3).

A multivariable model including predictors of COVID-19 anti-S antibodies among vaccinated HCWs is shown in Table 4. Antibody response was found to be higher in HCWs who were non-smokers, previously infected, received Oxford/AstraZeneca vaccine (AZD1222), and completed two doses of vaccine by 4.5, 10.5, 12.6, 95 times, respectively.

## 4. Discussion

Evaluation of the impact of COVID-19 vaccines on HCWs relies on understanding the kinetics of post-vaccine antibody response and identification of the socio-demographic and vaccine-related characteristics that determine the variability of this response. One of the most important demographic parameters that affect the antibody response is age. An inverted association between age and post-vaccination antibody response was observed in several studies due to diminished generation of T-cell and B-lymphocyte with age [9,14,15]. In the present study, younger age was not significantly associated with antibody production. However, higher seropositivity was observed in those younger than 40 years (88.7%) than older group (80.2%). This finding corresponds well with a study conducted in Turkey, which recorded higher antibody titers in the age group of 30-39 years with no significant association [16].

There is increasing evidence that COVID-19 infection has higher mortality among men, attributed to higher plasma levels of innate immune cytokines and induction of non-classical monocytes among males [17]. In addition, more robust production of post-vaccination anti-S neutralizing antibodies was associated with the female gender [18]. In the current study, there was no apparent difference in antibody positivity between males and females (84.4% versus 83.5%). This conclusion was also arrived at by the study conducted by Uysal et al. (2021) [16].

Assessment of BMI is essential in evaluating the post-vaccine antibody response, as obese individuals usually express angiotensin-converting enzyme 2 (ACE2) receptors abundantly in their adipose tissue, which act as functional receptors to the S1 subunit of RBD. Obese individuals are more prone to infection than individuals with a normal BMI. Moreover, there is impaired vaccine efficacy and quick disappearance of antibodies after vaccination in obese individuals, attributed to the aging of immune antibody-producing cells due to maintained low-grade inflammation and abnormal cytokine profile from fat cells in obese individuals [19,20]. Uysal et al. (2021) reported that almost half of those with high antibody titers had a normal BMI, and only 9.9% of them were obese [16]. When the relationship between BMI and the post-vaccine antibody response was assessed in the present study although not statistically significant, the highest anti-S seropositivity (89.5%) was observed among morbid obese HCWs, and the least was among normal BMI (76.9%). Interestingly, it was previously reported that obese individuals mount a vigorous initial antibody response after influenza vaccination. However, the antibody levels decline significantly compared with normal BMI individuals. Consequently, this initial rise in antibody titer was not considered protective as it should be maintained throughout the post-vaccination period [21].

Previous studies reported variable results on the effect of smoking on post-vaccine antibody response. Smokers usually have lower post-vaccination anti-S antibodies due to the negative effect of nicotine on activation of innate and acquired immunity [22,23,24]. In the present study, the anti-S seropositivity was observed to be significantly lower in smokers than in non-smokers, and a positive correlation between the smoking index and antibody response was observed. This finding agreed with Uysal et al. (2021), where higher percentages of those with antibody titer >250 U/mL were non-smokers (72.5%) [16].

Some studies reported higher post-vaccination antibody response in those previously infected with COVID-19. Furthermore, they indicated that a single dose of some vaccines might be enough in confirmed COVID-19 persons [25,26,27]. The present study’s findings correspond well with this assumption as the antibody positivity and median antibody titers were significantly higher in HCWs with a history of previous COVID-19 infection. Moreover, the antibody response had been associated with the frequency of infection and duration since diagnosis (>3 months). On the contrary, an Italian study reported no effect of a previous infection on the anti-S level [28]. Humoral antibodies are produced by short-lived plasma cells in secondary lymphoid organs. Therefore, they increase rapidly in the blood and decrease after the first three months following natural infection [29]. In parallel with the natural course of humoral response, post-vaccination antibody response in the current study increased with the longer duration since infection (>3 months).

Different SARS-CoV-2 vaccine candidates had reached the final stages for vaccine safety and were approved in various countries. The pros and cons of these vaccines regarding their immunogenicity and protection rate need to be better understood. Oxford/AstraZeneca vaccine (AZD1222) is a monovalent vaccine composed of a single recombinant, replication-deficient chimpanzee adenovirus vector encoding the S glycoprotein of SARS-CoV-2. It was associated with striking stronger ACE2 blocking antibody responses than other vaccines. In addition, the anti-S titer among participants receiving AstraZeneca was higher than those with Sinopharm (BBIBP-CorV) vaccine (inactivated vaccines) [30]. Similar to this previous report, the present study recorded that HCWs who received Oxford/AstraZeneca vaccine (AZD1222) had higher median anti-S titer (71.9 RU/mL) (230.08 BAU/mL) than those who received Sinopharm (58.0 RU/mL) (185.6 BAU/mL). Furthermore, they had higher anti-S seropositivity (*p* = 0.012).

The natural process of humoral immunity and post-vaccination antibody protection takes time to build. The WHO stated that the first dose of vaccines gives only partial protection, and this protection increases with the administration of the second dose [31]. In the current study, after the first dose, anti-S antibodies were detected in 61.5% of HCWs, reaching 92.3% after the second dose. All HCWs with COVID-19 infection prior to vaccination were seropositive after the first dose of vaccine, while only 46.4% of those who had no previous infection (*p* = 0.002). The same observation was reported by several studies where higher seroconversion rates and antibody titers (2–3 times) were recorded in previously seropositive individuals after a single dose of vaccine [32,33,34,35]. The present study showed that the post-vaccine humoral immune response peaked after 14 days from the second dose (90%), which was comparable to the study of Tanrıöver et al. (89.7%) [36].

## 5. Conclusions

The present study revealed that the antibody positivity among vaccinated HCWs had specific predictors as the previous history of COVID-19 infection, receiving Oxford/AstraZeneca vaccine (AZD1222), administration of two doses of vaccine, and duration since vaccination (≥14 days).

## Figures and Tables

**Table 1:** Anti-S response among vaccinated HCWs according to their demographic and clinical data.

		Anti-S Antibody Response		*p*-Value
		Positive	Negative	
		No. (%)	No. (%)	
Gender	Male	54 (84.4)	10 (15.6)	0.893
	Female	66 (83.5)	13 (16.5)	
Age in years	<40	55 (88.7%)	7 (11.3%)	0.172
	≥40	65 (80.2%)	16 (19.8%)	
Comorbidities				
Diabetes (*n =* 16)		12 (75%)	4 (25%)	0.291
Hypertension (*n =* 32)		26 (81.2%)	6 (18.8%)	0.641
Lung diseases (*n* = 4)		3 (75%)	1 (25%)	0.508
Cardiac diseases (*n* = 4)		4 (100%)	0 (0%)	0.999
Renal diseases (*n* = 1)		1 (100%)	0 (0%)	0.660
BMI				
Normal		20 (76.9%)	6 (23.1%)	0.677
Overweight		50 (83.3%)	10 (16.7%)	
Obese		33 (86.8%)	5 (13.2%)	
Morbid obesity		17 (89.5%)	2 (10.5%)	

**Table 2:** Anti-S response among vaccinated HCWs according to previous COVID-19 infection.

		Anti-S Antibody Response		*p*-Value
		Positive	Negative	
		No. (%)	No. (%)	
COVID-19 infection	Yes	45 (97.8%)	1 (2.2%)	*p* < 0.002
	No	75 (77.3%)	22 (22.7)	
Frequency of infection	Once	44 (100%)	0 (0%)	*p* = 0.044
	Twice	1 (50%)	1 (50%)	
Duration since diagnosis (months)	≤3	2 (66.7%)	1 (33.3%)	*p* = 0.065
	>3	43 (100%)	0 (0%)	

**Table 3:** Assessment of ani-S Ab response among vaccinated HCWs according to vaccine-related factors.

		Anti-S Antibody Response		*p*-Value
		Positive	Negative	
		No. (%)	No. (%)	
Type of vaccine	Oxford/AstraZeneca vaccine (AZD1222) (*n* = 109)	97 (88.9%)	12 (11.1%)	*p* = 0.012
		Median = 71.9 RU/ml		
	Sinopharm (BBIBP-CorV) (*n* = 31)	21 (67.7%)	10 (32.3%)	
		Median = 5 8.0 RU/ml		
	Others (*n* = 3)	2 (66.7%)	1 (33.3%)	
Number of received doses	one	24 (61.5%)	15 (38.5%)	*p* = 0.001
	Two	96 (92.3%)	8 (7.7%)	
Duration since last dose (days)	<14	21 (63.6%)	12 (36.4%)	*p* = 0.43
	≥14	99 (90%)	11 (10%)	

**Table 4:** Multiple stepwise exact logistic regression for predictors of COVID-19 anti-S among vaccinated HCWs.

Factors	*p*-Value	OR A	95% C.I for OR	
			Lower	Upper
Non-Smokers	0.048 *	4.5	1.6	20.6
COVID infection	0.017 *	10.5	1.8	35.9
Frequency of infection	0.021 *	4.2	1.7	22.9
Oxford/AstraZeneca vaccine (AZD1222)	0.042 *	12.6	2.2	69.5
Number of vaccine doses (2 doses)	0.001 **	95.0	20.9	115.3
Duration since vaccination	0.043 *	1.1	1.0	3.6

OR A: Adjusted Odds Ratio; * *p* < 0.05, ** *p* < 0.01.

## Data Availability

Not applicable.
